# Supplementary material for: MetaQTL: a package of new computational methods for the meta-analysis of QTL mapping experiments
Source: BMC Bioinformatics. 2007 Feb 8;8:49. doi: 10.1186/1471-2105-8-49 (PMC1808479; doi:10.1186/1471-2105-8-49)
Supplement: Additional File 7 — MetaQTL Package : jar file and tutorial. This Zip archive contains both the MetaQTL JAR file and the files of the tutorial. [file 1471-2105-8-49-S7.zip › org.inra.metaqtl/doc/index-files/index-11.html]

L-Index


|  |  |  |
| --- | --- | --- |
| **Overview** \| Package \| Class \| Use \| **Tree** \| **Deprecated** \| **Index** \| **Help** | |  |
| **PREV LETTER**   **NEXT LETTER** | **FRAMES**    **NO FRAMES**     **All Classes** |  |


A B C D E F G H I K L M N O P Q R S T U V W X Y Z 

---


## **L**

**label** - Variable in class org.metaqtl.TreeNode: The label of the cluster **Layer** - Class in org.metaqtl.graph: **Layer(double, double)** - Constructor for class org.metaqtl.graph.Layer: Creates a layer without any width and heigth constraints which upper left corner will be located at the point (x,y). **LAYER\_HSPACE** - Static variable in class org.metaqtl.graph.MetaGraphPar: The horizontal space between layers. **LAYER\_VSPACE** - Static variable in class org.metaqtl.graph.MetaGraphPar: The vertical space between layers. **leaf** - Variable in class org.metaqtl.TreeNode: The status of the node. **LEFT\_LABEL** - Static variable in class org.metaqtl.graph.ChromLayer: **LEFT\_LABEL** - Static variable in class org.metaqtl.graph.ChromShape: **LEGEND\_BOX\_CEX** - Static variable in class org.metaqtl.graph.MetaGraphPar: **LEGEND\_FONT** - Static variable in class org.metaqtl.graph.MetaGraphPar: **LEGEND\_GRAD\_HEIGHT** - Static variable in class org.metaqtl.graph.MetaGraphPar: **LEGEND\_GRAD\_WIDTH** - Static variable in class org.metaqtl.graph.MetaGraphPar: **LEGEND\_HSPACE** - Static variable in class org.metaqtl.graph.MetaGraphPar: **LEGEND\_PART\_HEIGHT** - Static variable in class org.metaqtl.graph.MetaGraphPar: **LEGEND\_PART\_WIDTH** - Static variable in class org.metaqtl.graph.MetaGraphPar: **LEGEND\_SCALE\_UNIT** - Static variable in class org.metaqtl.graph.MetaGraphPar: **legendLayer** - Variable in class org.metaqtl.graph.MetaGraph: **LGroup** - Class in org.thalia.bio.entity: Class Description Here **LGroup()** - Constructor for class org.thalia.bio.entity.LGroup: **LGroup(String, IBioEntity)** - Constructor for class org.thalia.bio.entity.LGroup: **LGROUP** - Static variable in interface org.thalia.bio.IBioEntityTypes: The integer code for Linkage Group entity **LGroupBean** - Class in org.thalia.bio.entity.bean: Class Description Here **LGroupBean()** - Constructor for class org.thalia.bio.entity.bean.LGroupBean: **LGroupBeanAdapter** - Class in org.thalia.bio.entity.adapter: Class Description Here **LGroupBeanAdapter()** - Constructor for class org.thalia.bio.entity.adapter.LGroupBeanAdapter: **LGroupPosition** - Class in org.thalia.bio.util: Class Description Here **LGroupPosition(double)** - Constructor for class org.thalia.bio.util.LGroupPosition: **LGroupPosition(double, double)** - Constructor for class org.thalia.bio.util.LGroupPosition: **load(Reader)** - Method in class org.thalia.bio.entity.factory.BioEntityFactory: **load(InputStream)** - Method in class org.thalia.bio.entity.factory.BioEntityFactory: **load(Reader)** - Method in class org.thalia.bio.entity.factory.MapChartFactory: TODO (non-Javadoc) **load(InputStream)** - Method in class org.thalia.bio.entity.factory.MapChartFactory: TODO (non-Javadoc) **load(Reader)** - Method in class org.thalia.bio.entity.factory.MapMakerFactory: **load(InputStream)** - Method in class org.thalia.bio.entity.factory.MapMakerFactory: **load(InputStream)** - Method in class org.thalia.bio.entity.factory.MapRawFactory: **load(Reader)** - Method in class org.thalia.bio.entity.factory.MapRawFactory: **load(Reader)** - Method in class org.thalia.bio.entity.factory.MapTabFactory: **load(InputStream)** - Method in class org.thalia.bio.entity.factory.MapTabFactory: **load(InputStream)** - Method in class org.thalia.bio.entity.factory.OntologyTabFactory: **load(Reader)** - Method in class org.thalia.bio.entity.factory.OntologyTabFactory: Term\_id : the term identifient. **load(InputStream)** - Method in class org.thalia.bio.entity.factory.XmlBioEntityFactory: **load(Reader)** - Method in class org.thalia.bio.entity.factory.XmlBioEntityFactory: **loci()** - Method in class org.thalia.bio.entity.LocusContainer: **loci()** - Method in interface org.thalia.bio.IBioLGroup: **locus** - Variable in class org.thalia.bio.entity.bean.LGroupBean: **Locus** - Class in org.thalia.bio.entity: **Locus()** - Constructor for class org.thalia.bio.entity.Locus: **Locus(String, IBioEntity)** - Constructor for class org.thalia.bio.entity.Locus: **LOCUS** - Static variable in interface org.thalia.bio.IBioEntityTypes: The integer code for Locus entity **LocusBean** - Class in org.thalia.bio.entity.bean: Class Description Here **LocusBean()** - Constructor for class org.thalia.bio.entity.bean.LocusBean: **LocusBeanAdapter** - Class in org.thalia.bio.entity.adapter: Class Description Here **LocusBeanAdapter()** - Constructor for class org.thalia.bio.entity.adapter.LocusBeanAdapter: **LocusContainer** - Class in org.thalia.bio.entity: Class Description Here **LocusContainer()** - Constructor for class org.thalia.bio.entity.LocusContainer: **LocusContainer(String, IBioEntity)** - Constructor for class org.thalia.bio.entity.LocusContainer: **locusNames** - Variable in class org.metaqtl.ChromCluster: Ths locus name index table **LocusPositionComparator** - Class in org.thalia.bio.util: Class Description Here **LocusProperties** - Class in org.metaqtl.util: **LocusProperties()** - Constructor for class org.metaqtl.util.LocusProperties: **lod** - Variable in class org.metaqtl.Qtl.QTLStats: The lod score of the QTL. **lodDecrease** - Variable in class org.metaqtl.Qtl.QTLSupport: The value of the LOD decrease used to obtain the CI. **longForm()** - Method in class org.metaqtl.main.CmdLineParser.Option

---


|  |  |  |
| --- | --- | --- |
| **Overview** \| Package \| Class \| Use \| **Tree** \| **Deprecated** \| **Index** \| **Help** | |  |
| **PREV LETTER**   **NEXT LETTER** | **FRAMES**    **NO FRAMES**     **All Classes** |  |


A B C D E F G H I K L M N O P Q R S T U V W X Y Z 

---
